# Prevalence and Clinical Relevance of *Schistosoma mansoni* Co-Infection with *Mycobacterium tuberculosis*: A Systematic Literature Review

**DOI:** 10.4236/ojepi.2023.131008

**Published:** 2023-02-23

**Authors:** Bocar Baya, Bourahima Kone, Amadou Somboro, Ousmane Kodio, Anou Moise Somboro, Bassirou Diarra, Fah Gaoussou Traore, Drissa Kone, Mama Adama Traore, Mahamadou Kone, Antieme Georges Togo, Yeya Sadio Sarro, Almoustapha Maiga, Mamoudou Maiga, Yacouba Toloba, Souleymane Diallo, Robert L. Murphy, Seydou Doumbia

**Affiliations:** 1 University Clinical Research Center (UCRC) of the University of Sciences, Techniques and Technologies of Bamako (USTTB), Bamako, Mali; 2 Service of Pneumopthisiology of the University Teaching Hospital of Point G, Bamako, Mali; 3 Clinical Laboratory of the University Teaching Hospital of Point G, Bamako, Mali; 4 Havey Institute for Global Health (Havey IGH), Northwestern University (NU), Chicago, USA

**Keywords:** *Schistosoma mansoni*, Tuberculosis, Co-Infections, LTBI, Reactivation

## Abstract

Tuberculosis disease stands for the second leading cause of death worldwide after COVID-19, most active tuberculosis cases result from the reactivation of latent TB infection through impairment of immune response. Several factors are known to sustain that process. *Schistosoma mansoni*, a parasite of the helminth genus that possesses switching power from an immune profile type Th1 to Th2 that favors reactivation of latent TB bacteria. The aim of the study was to assess the prevalence of the co-infection between the two endemic infections. Systematic literature was contacted at the University Clinical Research Center at the University of Sciences, Techniques, and Technologies of Bamako in Mali. Original articles were included, and full texts were reviewed to assess the prevalence and better understand the immunological changes that occur during the co-infection. In total, 3530 original articles were retrieved through database search, 53 were included in the qualitative analysis, and data from 10 were included in the meta-analysis. Prevalence of the co-infection ranged from 4% to 34% in the literature. Most of the articles reported that immunity against infection with helminth parasite and more specifically *Schistosoma mansoni* infection enhances latent TB reactivation through Th1/Th2. In sum, the impact of *Schistosoma mansoni* co-infection with *Mycobacterium tuberculosis* is under-investigated. Understanding the role of this endemic tropical parasite as a contributing factor to TB epidemiology and burden could help integrate its elimination as one of the strategies to achieve the END-TB objectives by the year 2035.

## 1. Introduction

Before the COVID-19 pandemic, *Mycobacterium tuberculosis* (*M. tb*) represented the deadliest infectious agent, killing more than 4000 people per day, exceeding the human immunodeficiency virus (HIV) and malaria two times each [1] [2] [3]. Despite, that tuberculosis (TB) incidence is slowly decreasing but still not sufficient enough to meet the World Health Organization’s (WHO) Ending TB objectives by the year 2035 [1] [4]. Latent TB infection (LTBI) is defined as an immunologically controlled *M. tb* infection with no apparent clinical and radiological sign of active TB. LTBI control involves the establishment of T helper 1 (type-1) inflammatory granuloma to block *M. tb* bacteria replication and spread. Several immune cells and cytokines are involved in this mechanism, such as T-cells lymphocytes, macrophages, monocytes, interleukin (IL)-1, 2, 6, 10, 12, IFN-*γ*, and TNF-*α* [5] [6] [7]. In immunocompetent individuals, LTBI can remain harmless throughout their entire lifetime. Reactivation or progression to active TB is defined as a transition from the latency stage to a symptomatic disease due to the escape and replication of the bacteria throughout a breach in the immune system induced by a new infection or failure to control an existing condition [5] [7] [8] [9]. Several health conditions can impair immune response leading to a possible reactivation of LTBI, they are classified from high-risk such as HIV, organ transplants, chronic hemodialysis, and alcohol abuse to low-risk factors including smoking, diabetes, and use of corticosteroids [10] [11].

*Schistosoma mansoni* (*Sch. m*) is one of the sub-species of *Schistosoma spp*, a parasite of the helminth family and trematode genus encountered in sleeping water and causing schistosomiasis, one of the neglected tropical infectious diseases (NTDs) still endemic in more than 78 resource-limited countries in Africa, South America, and Asia. Its prevalence in Sub-Saharan Africa represents 93% of all cases worldwide [12] [13].

**Figure 1.**
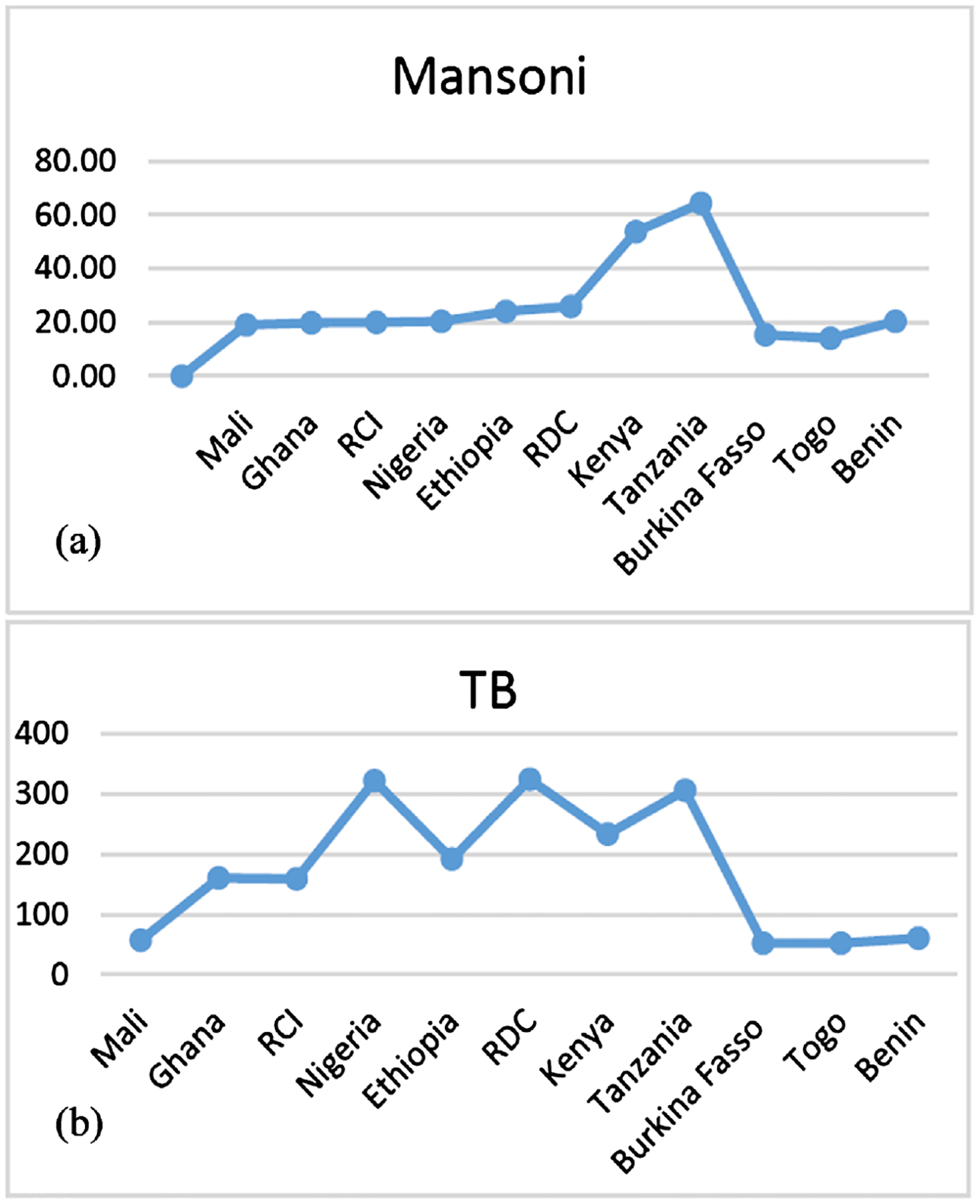
(a) Prevalence in percentage of Schistosomiasis in 11 Sub-Saharan African countries; (b) Incidence of Tuberculosis per 100,000 population in the same countries. Comparing the two figures, there is a trend between Schistosomiasis prevalence and Tuberculosis incidence.

Recent studies have shown that chronic infection with *Schistosoma mansoni* (*Sch. m*) impairs the host immune response and affects the formation and maintenance of type-1 granuloma by inducing T helper 2 (type-2) inflammatory granuloma. The process enhances a switch from the host’s existing type-1 immune response profile to a type-2 that weakens the strength of type-1 granuloma [14]. Cytokines produced during *Sch. m* chronic infection such as IL-5, 9, 10, and 13 were found to be significantly associated with liver fibrosis [15]. A decrease in CD4+ T cell response was observed in LTBI patients co-infected with helminth parasites which increased after treatment [16]. Similarly, a lower protective effect of the *Bacilli Calumet-Guerin* (BCG) vaccine in people with *Sch. m* infection which may lead to *M. tb* reactivation in case of LTBI co-infection [17]. Hematological and biochemical parameters were found to be altered in patients with *Sch. m* infection compared to healthy individuals who were improved after praziquantel treatment [18]. Several clinical studies reported statistically significant associations between the two infections and have also strengthened the hypothesis that *Sch. m* infection enhances LTBI reactivation to active TB through the reduction of the host’s protective immune response. A study in Uganda (2006) assessed the incidence of active TB in HIV patients co-infected with *Sch. m* versus HIV mono-infected and found an increased risk of developing active TB in HIV and *Sch. m* co-infected patients compared to those without *Sch. m* co-infection [19]. A case-control study in Tanzania (2017) also observed similar findings, patients with active TB had significantly higher odds of being *Sch. m* co-infected compared to their household healthy controls [20]. Based on pulled observation data from the WHO Global TB reports, there is a trend between Schistosomiasis endemic areas and TB burden. Active TB incidence is found to be higher in geographic regions where the prevalence of Schistosomiasis infection is important in sustaining the possible relationship between the two endemic infectious agents (Figure 1). The treatment of *Schistosoma mansoni* consists of a single oral dose of Praziquantel (PZQ) 40 mg/Kg repeated after 2 – 4 weeks. The first dose will kill adult worms present in the intestines and the second will target new parasites from egg hatch [21]. The treatment regimen recommended by WHO for people with drug-susceptible TB is a 6-month duration [22] including 2-month of isoniazid (H), rifampicin (R), ethambutol (E), and pyrazinamide (Z) followed by 4-month of isoniazid, rifampicin (2RHZE/4RH). Several regimens are proposed for LTBI treatment, from monotherapy with Rifampicin daily dose for 4-month or Isoniazid 6 or 9-month to bi-therapy with Isoniazid and Rifapentine weekly for 3-month or Isoniazid and Rifampin daily for 3-month [23]. Some works have been conducted on the immunological harm of the co-infection between tuberculosis and *Schistosoma mansoni* but still, there is more to be investigated for clinical evidence. The treatment of LTBI is confronted with several issues; insufficient investigations to differentiate active from latent TB and poor treatment adherence. The aim of this systematic literature review was to assess the effects of *Sch. m* infection on LTBI reactivation to active disease and existing data on the frequency of co-infections between *M. tb* and *Sch. m*.

## 2. Materials and Methods

### 2.1. Study Design and Setting

This study was designed to evaluate the clinical relevance of TB/*Sch. m* co-infection in the disease progression and active tuberculosis epidemiology dynamic. Therefore, published original research articles that reported the immunological interaction, and clinical frequencies of *Mycobacterium tuberculosis* and *Schistosoma mansoni* co-infections were searched and reviewed.

### 2.2. Article Search

Relevant research articles were searched using two search terms from two different databases, (Google Scholar, Embase, Scopus, PubMed, and Web All of Science) detailed as follow:

“*Mycobacterium tuberculosis*” [MeSH Terms] OR (“mycobacterium” [All Fields] AND “tuberculosis” [All Fields]) OR “*Mycobacterium tuberculosis*” [All Fields] OR (“tuberculosis” [All Fields] AND “mycobacterium” [All Fields]) OR “tuberculosis mycobacterium” [All Fields]) AND (“tuberculosis” [All Fields] OR “tuberculosis” [MeSH Terms] OR “tuberculosis” [All Fields] OR “tuberculoses” [All Fields] OR “tuberculosis” [All Fields]) AND (“*Schistosoma mansoni*” [MeSH Terms] OR (“schistosoma” [All Fields] AND “mansoni” [All Fields]) OR “*Schistosoma mansoni*” [All Fields]) AND (“co-infect” [All Fields] OR “co-infected” [All Fields] OR “co-infecting” [All Fields] OR “co-infection” [MeS Terms] OR “co-infection” [All Fields] OR “co-infections” [All Fields] OR “co-infects” [All Fields]).

All full-text articles found throughout this database search were assessed and all those eligible with an abstract in English were included in this study regardless of the language of the full article.

### 2.3. Inclusion and Exclusion Criteria

All full-text research articles from the search result of the study using the two search terms built from the words “Tuberculosis and *Schistosoma mansoni*” were included in the study. All studies that full text or abstract have not been found in the search and studies that do not include results of one of the two infections were excluded from the study.

### 2.4. Data Collection and Interpretation

Each included article was reviewed to retrieve information about TB and *Schistosoma mansoni* co-infections. Article on immune response interactions between the two-infection found in the literature was also described and analyzed in this literature review. Case reports, case series, case control, and cohort studies were summarized in different tables (Table 1 & Table 2).

### 2.5. Ethical Approval

The conduct of this systematic review was approved through a current project on the prevalence of *Schistosoma mansoni* among pulmonary TB patients in Mali by the Ethics committee of the Faculties of Medicine and Odonto-Stomatology, and Pharmacy of the University of Sciences, Techniques, and Technologies of Bamako (USTTB) in Point-G, Bamako, Postal Box: 1805; Mali.

## 3. Results and Discussion

### 3.1. Search Results

**Figure 2.**
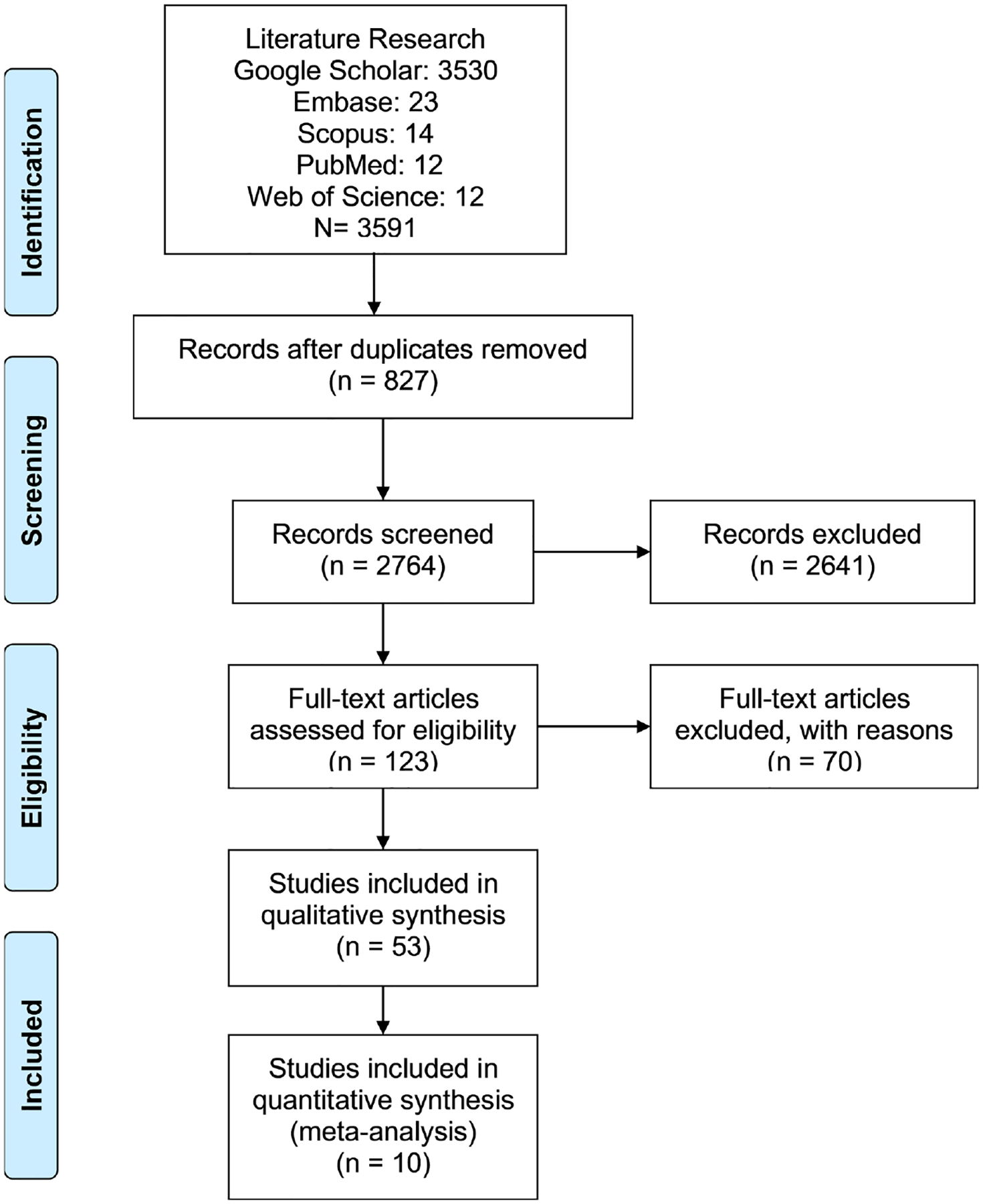
Data search flow chart. From: Moher D, Liberati A, Tetzlaff J, Altman DG, The PRISMA Group (2009). Preferred Reporting Items for Systematic Reviews and Meta-Analyses.

In total five (5) search engines were explored using the same term, three thousand five hundred ninety-one (3591) articles were found in the databases, including *Google Scholar* (3530), *Embase* (23), *Scopus* (14), *PubMed* (12) and *Web of Science* (12) (Figure 2). After removing duplicates and those not fulfilling TB and *Schistosoma mansoni* co-infection criteria, one hundred twenty-three (123) articles were eligible among those, fifty-three (53) were used for qualitative discussion and ten (10) for the quantitative analysis and data discussion.

### 3.2. *Mycobacterium tuberculosis* and Helminth Co-Infections: Expression of Th2 Immune Response Profile

The immunological disturbances occurring during *M. tb* and helminth co-infections have been reported in animal but also human studies. Since the beginning of the 20^th^ century, *Kullberg et al*. (1992) observed that *Sch. m* infection down-regulates the host’s Th-1 immune response, and induces an exaggerated Th-2 response that alters existing protective immune response against other infectious agents; the findings were later supported by observations of *Rafi et al*. (2012), that helminth infection diminishes immune protection against LTBI [24] [25] [26]. In vaccinology, helminth chronic infection has also been found to affect immunization conferred by vaccines; *Kondĕlková et al*. (2010) & *Méndez-Samperio et al*. (2014), found in their investigation that Treg cells modulate immune reaction by suppressing cell’s multiplication and production of cytokines, implying that the presence of helminth infection confers a buffer medium that neutralizes the effect of ongoing immune responses [27] [28]. *McArdle et al*. (2018) reported that reactivation of latent TB infection (LTBI) in the presence of helminth infection comes from a switch from Th1 to Th2 cytokine production [29]. *Abate et al*. (2015) observed that the simple presence of helminth infection without any clinical manifestation in patients with active TB could downregulate host immune response similar to that seen in patients with immunodepression. The co-infection creates a coexistence of T-Regulatory Cells (Treg cells), and type-1 cytokines (IL-10) but also the secretion of type-2 expressing cytokines (IL-5) conferring an immunodeficiency status to the patient. Thereby, the low bacteria load in sputum observed in TB and *Sch. m* co-infected patients was already reported as a common finding in TB and HIV co-infection [30] [31]. *Babu et al*. (2016) also brought evidence from the animal model and human studies that helminth infections affect the host immune control of TB infection, thereby increasing the risk of TB bacteria reactivation in an organism with both infections [32]. *Rajamanickam et al*. (2019 & 2020) found that helminth and LTBI co-infection exposes LTBI control failure by inducing low-production chemokines and alteration of their function. They also observed an alteration of monocyte cells’ function through inadequate activation and polarization during the co-infection [33] [34]. C*admus et al*. (2020) reported in a literature review that helminth infection negatively impacts the strength of vaccination response, thereby immunization programs should consider helminth endemic areas regarding the protection level of vaccines [13] *Kiflie et al*. (2021) found that helminth co-infection in active TB patients had a positive correlation between TB disease severity and an increase of helminth-specific TGF-*β*+ Tregs that was restored after anti-helminth treatment [35]. *Resende et al*. observed that TB and helminth co-infection leads to a decrease in T-cells and natural killers number, cytokine production, and severe lung damage compared to TB mono-infected patients and healthy controls [36] [37]. A similar observation was reported by *Bogdan et al*. (1991); *Gong et al*. (1996); *Abate et al*. (2015) and *Aira et al*. showed that IL-10 production down-regulates Th1-driven immune response against TB infection [38] [39] [40] [41]. These observations demonstrate that in the presence of helminth infection, the expression of the Th2 immune profile involves Treg cell proliferation and certain types of cytokines that down-regulate existing Th1 immune inflammatory response against other types of infections and particularly in the case of LTBI.

### 3.3. *Schistosoma mansoni* Impairs Immune Response to *Mycobacterium tuberculosis* Leading to Latent Tuberculosis Infection Reactivation

In more than four past decades, *Olds et al*. (1981) reported that *Sch. m* co-infection impairs immune response in active TB patients, and a decrease in killer monocyte cell rate was observed in *Sch. m* co-infected and TB patients [42]. Several other studies supported the hypothesis that in the presence of *Sch. m* infection, the host immune response undergoes a modulation process leading to a poor immune response quality against a new or an existing infection. *Elias et al*. (2006) reported a low protective effect of the BCG vaccine in children infected with *Sch. m* [17]. The observation was further supported by *Musaigwa et al*. (2022) that *Sch. m* infection affects vaccine response again TB infection by inducing deaths of plasmablast and plasma cells in the bone marrow [43]. Among the diverse helminth, *Sch. m* one of the Schistosoma subspecies, has been identified to possess specific proteins that lead to failure of maintaining type1 granuloma formation by promoting a strong type 2 inflammatory immune response. *Monin et al*. (2015) were pioneers of that observation by describing that during *M. tb* and *Sch. m* co-infection, *Sch. m* increases susceptibility to TB reactivation but also the disease severity by increasing the level of inflammatory response with the accumulation of arginine-1 expressing macrophages [44]. *Pearce et al*. also demonstrated that the presence of soluble *Sch. m* egg antigen inhibits IL-12 cytokine production by dendritic cells and induces a Th2 inflammatory immune response profile promoting T-cell regulatory (Treg) responses that inhibits the establishment of type Th1 response [45]. *Giera et al*. have also identified lipids such as prostaglandins (rich in *Sch. m* eggs) to be specifically driving expression of type Th2 immune response profile during *Sch. m* infection [46]. *Meurs et al*. in Senegal investigated cytokine production during schistosomiasis infection and observed that *Sch. m* induces the production of Th2 profile cytokines but *is* not as stronger compared to *Schistosoma haematobium* (*Sch. h*). This implies that *Sch. m* can induce a switch of immune response profile from Th1 to Th2 but does not induce such a stronger response to limit damages from other infections [47]. *Schramm et al*. (2018) investigated the immune modulatory effect of *Sch. m* and observed that using *Sch. m* egg-specific antigens can significantly decrease the immune response against Salmonella infection [48]. *Dinardo et al*. (2016) observed that *M. tb*-specific CD4+ T-cell functions are altered in presence of soluble schistosome antigen and block the maturation of macrophage phagolysosome [49]. However, *McLaughlin et al*. (2020) reported a contradictory finding that Th1 immune functions are still maintained by TCD4 cells during TB and *Sch. m* co-infection [50].

**Figure 3.**
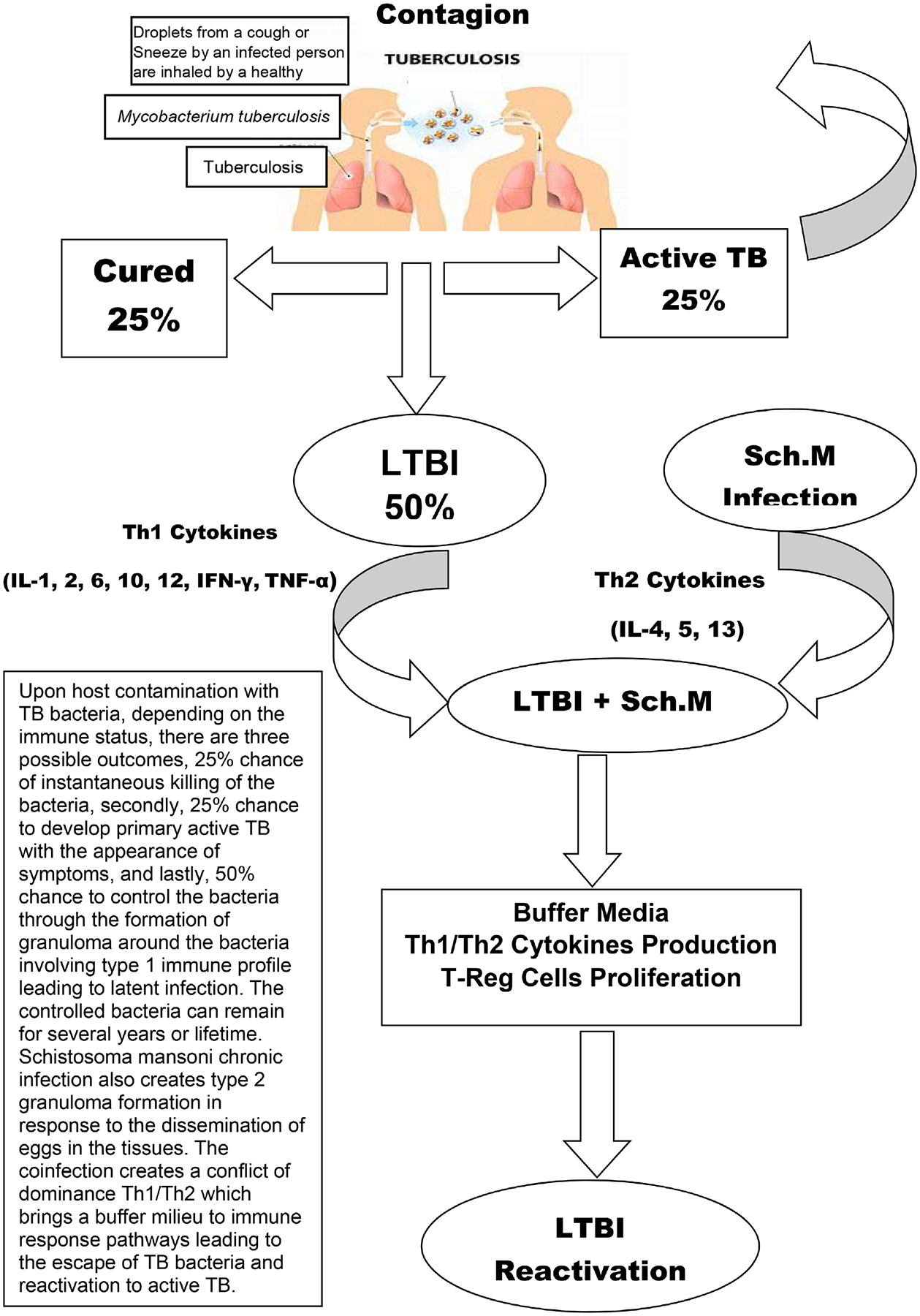
TB Infection Transmission and reactivation through *Schistosoma mansoni* infection.Th1/Th2 immune impairment during *Schistosoma mansoni* Latent TB co-infection.

Lessons learned from this step are that *Sch. m* infection induces a buffered media in the immune response pattern and plays an important role in TB infection control. In the presence of *Sch. m* antigens, the host immune system fails to establish an appropriate defense against other infections. This immunological disaster happens when the host immune system is facing an acute infection and knocking it down to a latency phase or when *Sch. m* infection occurs in an individual with an immunologically well-controlled latent TB infection (Figure 3).

### 3.4. Prevalence of *Schistosoma mansoni* and Tuberculosis Coinfections across Clinical Studies

*Schistosoma mansoni* and *Mycobacterium tuberculosis* co-infections have gained increasing interest over the last two decades. Observations started with single case reports; *Cristina et al*. (2006), in Brazil reported a case of hepatic TB co-infected with *Sch. m* [51]. *Basile et al*. (2007) published one of the first cases of *Sch. m* and *M. tb* tuberculosis co-infection in a 32-year-old, male, living in France but of Sub-Saharan African origin [52]. *Gobbi et al*. (2014), further support a case of *Schistosoma mansoni* and *Mycobacterium tuberculosis* co-infection mimicking a single infection of *Schistosoma mansoni* in the lung [53]. *Range et al*. (2007) in Tanzania found more than one-third (35.5%) of *Schistosoma mansoni* co-infection among confirmed pulmonary TB patients and also HIV co-infection was 43.6% [54] Ten years later, structured case series started to report remarkable frequencies, and clinical characteristics of patients with the co-infection. Earlier in 2012, *Abate et al*. (2012) conducted another study in Ethiopia, the frequency of *Sch. m* co-infection among TB patients was 19% [55]. *Li & Zhou* (2013) conducted a systematic review on TB and parasite co-infections, they observed a prevalence of 5.4% of *Sch. m* among TB patients [56]. *McLaughlin et al*., in a recent study in Kenya, it has been observed a 4% increase in TB incidence in HIV-Negative people infected with *Sch. m* (19.7%) compared to *Sch. m*-uninfected cases (15.8%) whereas a 14% increase was observed in TB incidence in HIV-Positive patients co-infected with *Sch. m* (27.3%) compared to HIV-Positive *Sch. m*-uninfected patients (41.2%) [57]. *Tegegne et al*. (2018) conducted a study in Gondar (Ethiopia) that reported a prevalence of 0.4% of *Sch. m* in patients suspected of pulmonary tuberculosis [58]. *Gashaw et al*. (2019) reported a prevalence of 4.3% of *Sch. m* in active TB patients with under-nutritional status in Northeastern Ethiopia [59]. *Mhimbira et al*. (2017) found 5.7% of *Sch. m* co-infection in TB patients compared to their household control individuals. There was a 2.15-fold higher risk of *Sch. m* co-infection in TB patients compared to their household controls. *Sch. m* and M.tb co-infected patients had a 2.63-fold risk of being in the group of TB patients with lower sputum bacterial load, and also 0.41-fold less chance of having pulmonary cavitations [20]. *Sikalengo et al*. (2018) in Tanzania found 16.4% of *Sch. m* co-infection among active TB patients living in the rural area of Dar Salam [60]. From the findings of this review, there is evidence of an association between the two infections that *Sch. m* infection increases active TB incidence in individuals with LTBI regardless of their immunological status and there is a clear pathway explaining the mechanism of switching Th1 immune response to a predominant Th2 cytokines production creating a buffer media that drive out the immune control of TB latent bacteria.

## 4. Conclusions

This Systematic Literature Review has narrowed down findings from different investigational studies on the harms of *Sch. m* infection on the acquired immune response that controls *M. tb* infection during the latency stage.

Th2-driven immune response expressed in *Sch. m* infection down-regulates Th1-induced immune reaction which disrupts immunological cascades against other infectious agents, particularly in patients with LTBI where cell-based immune reaction is predominantly needed to lockdown the TB bacteria in a latent stage when failing its elimination.

Based on this review, we speculate that strategies for TB elimination must consider *Schistosoma mansoni* eradication actions where both infections are endemic. Large sample sizes and multicentric cohort studies are needed to deeply investigate the epidemiological and clinical implications of *Sch. m* infection on the global active TB burden, especially in endemic settings.

## Figures and Tables

**Table 1.** Case reports of co-infection between TB and *Schistosoma mansoni*.

Country	Year of report	TB localization	*Sch. m* & Parasitic disease	Sex	Age (yrs)	HIV test	Patient origin	No. of reference
Australia	2001	Pulmonary tuberculosis	*Schistosoma Japonicum*	M	30	Unknown	Philippines	*Torresi etal*. [61]
Brazil	2006	Hepatic tuberculosis	*Schistosoma mansoni*	Male	17	Unknown	Brazil	*Ferrari et al*. [51]
France	2007	Lymphadenitis tuberculosis	*Schistosoma mansoni*	Male	32	Unknown	Mauritania	*Basile et al* [52]
Italy	2014	Pulmonary tuberculosis	*Schistosoma mansoni*	Male	27	Negative	Mali	*Gobbi et al*. [53]

**Table 2.** Frequency of *Mycobacterium tuberculosis* and *Schistosoma mansoni* co-infections.

Country	Year of report	Sample Size (n)	TB patients screened (n)	TB/Sch. *m* co-infection (n (%)	Control patients (n)	*Sch. mansoni* co-infection (%)	Odds Ratio 95% CI (aOR), p-value	Reference
Uganda	2006	462	462	10.0	20/168		2.31 (1.0 – 5.3)	*Brown et al*. [19]
Tanzania	2007	655	532	34				*Range et al*. [54]
Ethiopia	2012	112	32	19.0	38	16.5		*Abate et al*. [55]
Tanzania	972	2017	597	5.7	375	4.0	2.15 (1.0 – 4.5) p = 0.040	*Mhimbira et al* [20]
Tanzania*	2018	668	668	7.9				*Sikalengo et al* [60]
Ethiopia	2019	384	384	4.3				*Gashaw et al*. [59]
Kenya^□^	2021	941	194	14.0	747	4.0		*McLaughlin et al*. [57]

Tanzania*: *Schistosoma mansoni* was 16.4% in the rural area vs. 4.13% in the Urban. Kenya □: HIV/TB co-infected patients.
